# Potential regulatory role of miRNA and mRNA link to metabolism affected by chronic intermittent hypoxia

**DOI:** 10.3389/fgene.2022.963184

**Published:** 2022-09-06

**Authors:** Yanru Duan, Shihan Zhang, Yu Li, Wen Zhao, Pinxue Xie, Xi Zhang, Yunhui Du

**Affiliations:** ^1^ Department of Physiology and Pathophysiology, School of Basic Medical Sciences, Capital Medical University, Beijing, China; ^2^ Beijing Key Laboratory of Pediatric Hematology Ocology, Key Laboratory of Major Diseases in Children, Pediatric Oncology Center, National Center for Children’s Health, Ministry of Education, Medical Oncology Department, Beijing Children’s Hospital, Capital Medical University, Beijing, China; ^3^ Center for Coronary Artery Disease, Department of Cardiology, Beijing Anzhen Hospital, Capital Medical University, Beijing, China; ^4^ Beijing Institute of Heart Lung and Blood Vessel Disease, Beijing Anzhen Hospital, Capital Medical University, Beijing, China

**Keywords:** obstructive sleep apnea, chronic intermittent hypoxia, adipocyte, miRNA, mRNA

## Abstract

**Aim:** Intermittent hypoxia (IH) is the prominent feature of obstructive sleep apnea (OSA) pathophysiology, which is an in dependent risk factor of cardiovascular complications. The effects of IH on adipocyte metabolism were explored by high-throughput sequencing technology.

**Methods:** Plasma was collected from OSA patients and control group to perform mRNA sequencing. 3T3-L1 cells were differentiated into adipocytes then subjected to a 5%–21% O_2_ hypoxic environment (IH) for 24 h. High-throughput sequencing method was used to determine differential mRNA and miRNA patterns in fat cells exposed to IH. We then performed Gene Ontology (GO) analysis, identified relevant KEGG pathways and miRNA-target-pathways.

**Results:** Sequencing data showed that OSA affected the expression of 343 mRNAs in the plasma. At the same time, we found that IH affected the expression of 3034 mRNAs in the adipocytes. In addition, 68 differentially expressed mRNAs were overlapped in plasma from OSA patient and IH-induced adipocyte model. We observe that 68 differential genes could be connected to 49 reciprocally expressed miRNAs. We showed that IH significantly reduced the expression of miR-182-5p and miR-30c-2-3p. KEGG predicted that the function of expressed miR-182-5p and miR-30c-2-3p was enriched to AKT signaling pathway. Notably, IH activated PI3K/AKT pathway in fat cells.

**Conclusion:** Our results demonstrated that IH might induce adipocyte metabolism by regulating miR-182-5p and miR-30c-2-3p.

## Introduction

Obstructive sleep apnea-hypopnea syndrome (OSA), characterized by recurrent upper airway collapse during sleep, causes multiple physiological perturbations (Yeghiazarians et al., 2021). Recent research has reported that OSA is an independent risk factor and is associated with significant cardiovascular morbidity and mortality (Mazzotti et al., 2019). Many clinical and research studies have provided evidence for a correlation between OSA and metabolic dysfunction (Gileles-Hillel et al., 2016). Chronic intermittent hypoxia (IH) is a main feature of OSA and is traited by repeated circulating reoxidation and hypoxemia. Recent research has reported that OSA induces cardiovascular diseases through IH (Yeghiazarians, et al., 2021). More and more evidence supported that OSA/IH increases the risk of metabolic dysfunction in adipose tissue (Ryan et al., 2019). The underlying mechanisms remain incompletely understood.

Adipose tissue is an important regulator of whole-body metabolism and energy balance (Felix et al., 2021). Adipocytes arising from mesenchymal stem cells in adipose tissue (MSCs) sustainably differentiate into mature adipocytes (Gentile et al., 2020). Adipocytes are potent producers of proinflammatory cytokines and secrete high amounts of adipokines that regulate metabolism, vascular and endothelial function and many other body processes. It has been reported that lactate is increased in hypoxic adipocytes with corresponding increases in glucose utilization (Ota et al., 2019). Evidently, hypoxia induces insulin resistance in adipocytes and brings about adipose tissue dysfunction (Trayhurn, 2013). Such adipocyte dysfunction in OSA is related to cardiovascular disease (CVD) morbidity and mortality (Yeghiazarians, et al., 2021). However, our understanding of the underlying mechanisms of OSA effects on adipocytes is still limited.

Recent efforts have been made to identify novel biomarkers and early diagnostic methods for the onset and progression of metabolic disorders (Su and Peng, 2020). miRNAs are involved in gene expression, cell differentiation, and signal transduction *via* the recognition of cognate sequences and modulation of transcriptional, translational or epigenetic processes (Lu et al., 2019). In recent years, the underlying molecular mechanisms of metabolic disease have become critical to understanding their pathophysiology (Fan and Pedersen, 2021). However, there has been no comprehensive genome-wide analysis of IH-induced metabolic regulation in adipocytes. Therefore, in this study, we have started to address this gap by performing genome-wide transcriptional and miRNA sequence analyses to better understand how IH-triggered metabolic changes affect adipocyte cells.

## Materials and methods

### Participants

Participants were recruited from Anzhen Hospital, Capital Medical University, Beijing, China from February 2017 to December 2017. The clinical characteristics of OSA patients are summarized in Supplementary Table S1. The study has been authorized and registered by Medical Ethics Committee of Beijing Anzhen Hospital (2017005) together with China Clinical Trial Registry (CHICTR-ROC-17011027) and written informed consents were obtained from all the participants. OSA patients were diagnosed on the basis of American Academy of Sleep Medicine Guidelines for an apnea-hypopnea index (AHI) ≥ 5 per hour. Exclusion criteria were other sleep disorders, upper airway resistance syndrome, acute infectious disease, renal disease, and hepatic disease. Venous blood samples were collected at admission without anticoagulant. After centrifugation at 4°C, the plasma was immediately separated and stored at −80°C until further analysis.

### Cell culture and chronic intermittent hypoxia treatment

Mouse 3T3-L1 cells were first cultured in DMEM-high glucose supplemented with 1% penicillin/streptomycin and 10% fetal bovine serum (FBS). We induced differentiation into adipocytes by exposing cells for 2 days to induction media consisting of DMEM-basal medium, 0.1–0.4 μM insulin, 0.25 μM dexamethasone, 0.5 mM isobutylmethylxanthine, and 1 μM rosiglitazone. After 2 days, this was changed to maintenance growth media consisting of DMEM-basal medium, and 0.1 μM insulin. The maintenance media were fully changed every other day until day 8. After 8–12 h, the media was then changed back to maintenance growth media.

IH was induced by using a BioSpherix OxyCycler C42 system (BioSpherix, Redfield, NY, United States) cycling 5%–21% O_2_ per hour. Adipocytes were cultured to either IH or normoxic conditions (NOR, continuous 21% O_2_ as a control) over 24 h.

### miRNA sequencing

Total miRNA was collected from fat cells using mirVnanTM miRNA kit (AM1561, Thermo Fisher Scientific, United States) and purified using an RNeasy mini kit (Qiagen, Valencia, CA, United States). A miRNA Library was generated using the HiSeq 2500 system according to the manufacturer’s instructions. Quality controls were performed and miRNA libraries were then sequenced at 10 p.m. on the Illumina HiSeq 2500 to generate 150 bp paired-end reads. *p*-values were corrected using false discovery rate (FDR) and considered significant at *p* < 0.05 and > 2fold change.

### miRNA gene ontology and pathway enrichment analysis

We performed GO analysis [Biological Process (BP), Molecular Function (MF) and Cellular Component (CC)] to identify the function of the differentially expressed miRNAs. Then we used pathway analysis to identify significant functional implications of the differential genes according to the Kyoto Encyclopedia of Genes and Genomes (KEGG) database. In all instances, we used the two side Fisher’s exact test and the Benjamini–Hochberg procedure to correct for multiple tests correction. We set the statistical significance thresholds at *p* < 0.01 for GO analyses and *p* < 0.05 in our pathways analyses.

### RNA sequencing

Total RNA from plasma and adipocyte was extracted using TRIzol (Invitrogen). The RNA was reverse transcribed into double strand cDNA fragments. The RNA Library was generated using a NEB Next Ultra Directional RNA Library Prep Kit for Illumina (#E7530L, NEB, Ispawich, United States) per the manufacturer’s protocol.

After the final library quality controls, HTseq2500 was used to count the read count for each gene in each sample, and the expression level of genes in each sample was shown by FPKM (Fragments Per Kilobase Millon Mapped Reads). DESeq2 v1.20.0 (http://bioconductor.org/packages/release/bioc/html/DESeq2.html) (Anders and Huber, 2010) analyzes differential expression for RNA seq. The Benjamini & Hochberg method, a technique adept at controlling false discovery rate, is used to correct *p*-values (Benjamini and Hochberg, 1995). Briefly, all *p* values are placed in order, from smallest to largest. The smallest *p* value received a rank i = 1, the next = 2, and so on. Next, we compared each *p* value to its Benjamini–Hochberg critical value, (i/m) Q, where i is the rank, m is the total number of tests, and Q is the false discovery rate you choose. Finally, identifying the largest *p* value that is smaller than the Benjamini–Hochberg critical value is assigned as significant at the relevant threshold. A default corrected *p*-value (FDR) of 0.05 was set as the significantly differential expression.

### miRNA target-pathway/gene network

The miRNA target-pathway network and miRNA target gene network were built according to the relationships among miRNAs and pathways/genes. We visualized the networks using Cytoscape (vension:3.6.0). In the miRNA target-pathway/miRNA target-gene networks, pathways/genes are represented by circles, miRNA by squares, and relationships by lines. The number of pathways/genes regulated by that miRNA determined the degree of each miRNA, and the number of miRNAs that regulated the pathway/genes also determined the degree of each pathway/gene. Those that had the largest degrees were key miRNAs and pathways/genes in the network.

### Real-time quantitative PCR

Reverse transcription and qPCR were conducted by using Hairpin-it MicroRNA RT-PCR Quantitation Kit (GenePharma, China). Briefly, the first-stand cDNA was synthesized *via* Hairpin-it MicroRNA RT-PCR Quantitation Kit. qRT-PCR was performed utilizing Real-time PCR Mastermix on the 7500 Real-Time PCR system (Thermo Fisher Scientific, Inc.). Sequences for qRT-PCR primers are shown below:

mir-182-5p:5′-CTGCTGTTTGGCAATGGTAGA-3′;

mir-30c-2-3p: 5′-GGG​GTG​TAA​ACA​TCC​TAC​ACT​C-3′;

U6 snRNA: 5′-CGC​TTC​GGC​AGC​ACA​TAT​AC-3′.

### CCK8 assay

CCK-8 kit (Dojindo, Shanghai, China) was used to measure proliferation of 3T3-L1 cells. 1,000 cells per well in a volume of 100 μl were cultured in ten replicate wells in a 96-well plate in medium containing 10% FBS. Then the CCK-8 reagent (10 μl) was added to 90 μl DMEM to generate a working solution, of which 100 μl was added per well and incubated for 1 h. The optical densities were measured at a spectral wavelength of 450 nm using a microplate reader (Thermo Fisher Scientific, Inc.).

### Determination of the supernatant concentrations of APN

To assess adiponectin secretion in 3T3-L1 cells, the culture supernatant was harvested to measure adiponectin concentrations using an enzyme-linked immunosorbent assay according to the manufacturer’s instruction (Cat #8B5D6EB29B, Cloud-Clone Corp). The detection threshold was 0.156 ng/ml. Measurements were performed in eight replicates. Results were presented as ng/ml.

### Statistical analysis

Data are showed as mean ± SEM. Groups were compared using t-tests or one-way ANOVA with post hoc Bonferroni and Tukey tests, as appropriate. Statistical analyses were conducted using GraphPad Prism 8.0 (GraphPad Software Inc., San Diego, CA, United States) and SPSS 25.0 (SPSS Inc., Chicago, IL, United States) software.

## Results

### Plasma gene expression profile in obstructive sleep apnea patients

Plasma was collected from 40 subjects (OSA vs. Control, 20 vs. 20) to conduct RNA sequencing in this study. Baseline clinical and biochemical characteristics are presented in Supplementary Table S1. Compared with control group, the OSA patients had a higher BMI (28.13 ± 3.38 vs. 24.14 ± 2.81 kg/㎡, *p* < 0.001). The OSA group exhibited severe degrees of AHI (52.3 ± 20.2 vs. 3.2 ± 1.13, *p* < 0.001), significantly lower SpO2 (76.9 ± 10.4 vs. 88.8 ± 1.3, *p* < 0.001) in comparison with the control group.

**FIGURE 1:**
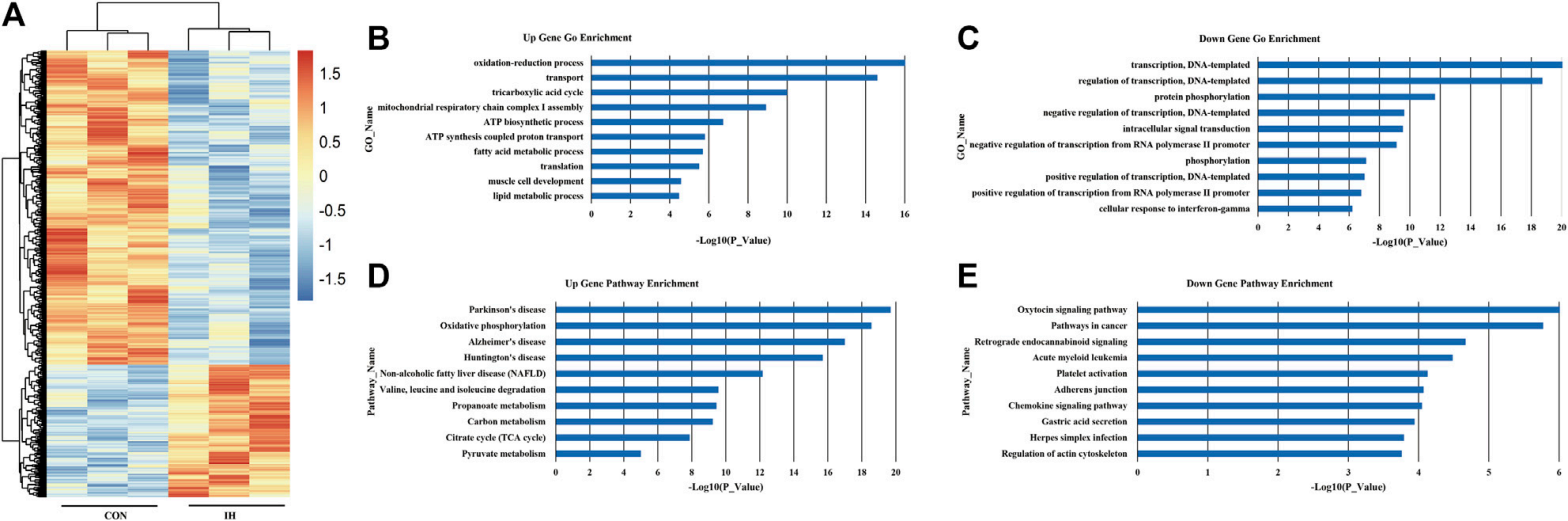
mRNA expression profile in plasma from OSA patients. **(A)** Differential expression of mRNA; **(B)**. Upregulated genes. **(C)** Downregulated genes. **(D)** Upregulated significant pathways. **(E)** Downregulated significant pathways. The y-axis shows the GO category and the x-axis shows a negative log-transformed *p*-value -lgP where a larger -lgP indicates a smaller *p*-value.

A hierarchical cluster analysis was performed to explore changes in transcriptional gene expression patterns between OSA group and control (Figure 1A). OSA was associated with 343 differentially expressed genes; of these, 58 genes were highly expressed, and 285 had low expression. To better understanding the biological functions of mRNAs, we performed GO and KEGG pathway enrichment analysis. The top 10 enriched GO terms of upregulated DEGs induced by OSA were oxidation-reduction process, transport, tricarboxylic acid cycle, mitochondrial respiratory chain complex I assembly, ATP biosynthetic process, ATP synthesis coupled proton transport, fatty acid metabolic process, translation, muscle cell development and lipid metabolic process (Figure 1B). The 10 major downregulated GO terms after OSA patients were related to transcription, DNA-templated, regulation of transcription, DNA-templated, protein phosphorylation, negative regulation of transcription, DNA-templated, intracellular signal transduction, negative regulation of transcription from RNA polymerase II promoter, phosphorylation, positive regulation of transcription, DNA-templated, positive regulation of transcription from RNA polymerase II promoter and cellular response to interferon-gamma (Figure 1C).

Based on the KEGG database, the top 10 significantly upregulated pathway induced by OSA were Parkinson’s disease, oxidative phosphorylation, Alzheimer’s disease, huntington’s disease, non-alcoholic fatty liver disease (NAFLD), valine, leucine and isoleucine degradation, propanoate metabolism, carbon metabolism, citrate cycle (TCA cycle) and pyruvate metabolism (Figure 1D). And the top 10 significantly downregulated pathways were oxytocin signaling pathway, pathways in cancer, retrograde endocannabinoid signaling, acute myeloid leukemia, platelet activation, adherents junction, chemokine signaling pathway, gastric acid secretion, herpes simplex infection and regulation of actin cytoskeleton (Figure 1E).

### Intermittent hypoxia-exposed adipocyte gene expression profile

**FIGURE 2:**
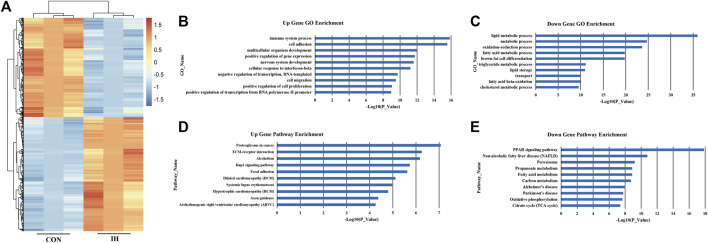
IH-exposed adipocyte gene expression profile. **(A)** Differential expression of mRNA; **(B)**. Upregulated genes. **(C)** Downregulated genes. **(D)** Upregulated significant pathways. **(E)** Downregulated significant pathways. The y-axis shows the GO category and the x-axis shows a negative log-transformed *p*-value -lgP where a larger -lgP indicates a smaller *p*-value.

To identify how chronic intermittent hypoxia (IH) affects mRNA expression patterns in adipocytes, 3T3-L1 cells were cultured to IH conditions over 24 h to perform RNA-sequencing analysis. The data show that IH was associated with 3034 differentially expressed genes; of these, 2278 genes were highly-expressed, and 756 had low expression (Figure 2A). The 10 major upregulated GOs induced by IH were related to immune system processes, cell adhesion, multicellular organism development, positive regulation of gene expression, nervous system development, cellular responses to interferon-beta, negative regulation of transcription, DNA-templated, cell migration, positive regulation of cell proliferation, and positive regulation of transcription from RNA polymerase II promoter (Figure 2B). The 10 major downregulated GOs after IH exposure were related to lipid metabolic processes, metabolic processes, oxidation-reduction processes, fatty acid metabolic processes, brown fat cell differentiation, triglyceride metabolic processes, lipid storage, transport, fatty acid beta-oxidation, and cholesterol metabolic processes (Figure 2C).

Based on the KEGG database, the top 10 significantly upregulated pathway interactions induced by IH were Proteoglycans in Cancer, ECM-receptor interaction, Alcoholism, Rap1 signaling pathway, Focal adhesion, Dilated cardiomyopathy (DCM), Systemic lupus erythematosus, Hypertrophic cardiomyopathy (HCM), Axon guidance, and Arrhythmogenic right ventricular cardiomyopathy (ARVC) (Figure 2D). And the top 10 significantly downregulated pathways were the peroxisome proliferator-activated receptor (PPAR) signaling pathway, Non-alcoholic fatty liver disease (NAFLD), Peroxisome, Propanoate metabolism, Fatty acid metabolism, Carbon metabolism, Alzheimer’s disease, Parkinson’s disease, Oxidative phosphorylation, and Citrate cycle (TCA cycle) (Figure 2E).

### miRNA-gene-network and miRNA-pathway-network based on the effects of intermittent hypoxia

**FIGURE 3:**
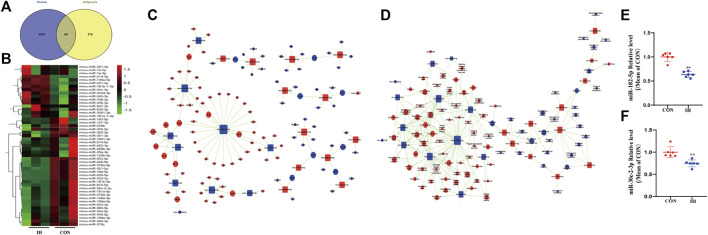
miRNA-Gene-Network and miRNA-Pathway-Network. **(A)** Venn diagram showed the number of overlapped genes of differentially expressed in adipocyte model and plasma from OSA patient. **(B)** Heatmap showed the 49 differentially expressed miRNAs related to expressed mRNAs by miRNA sequence analysis. **(C,D)** miRNAs are represented by Box nodes, and the predicted target genes/target pathways are represented by cycle nodes. The inhibitory effect of miRNA on its predicted targets is showed by Edges. The contribution of one miRNA to the surrounding genes or the contribution of one gene to the surrounding miRNAs are showed by Degrees. The miRNAs and genes/pathways always have the largest degrees in the network. **(E,F)** qRT-PCR analysis of key miRNAs in the effect of IH (**p value < 0.05).

According to the standard of 2-fold cutoff and *p* value less than 0.05, 68 differentially expressed mRNAs were overlapped in plasma from OSA patient and IH-induced adipocyte model (Figure 3A). To identify how chronic intermittent hypoxia (IH) affects miRNA expression pattern in adipocytes, IH-induced adipocytes were collected to perform miRNA sequence analysis. The upstream miRNAs of 68 differentially expressed genes were searched adversely by miRNA sequence data. 49 identical miRNAs were found in miRNA sequence (Figure 3B).

An intersection was got between the targets of miRNAs and differentially expressed genes. The miRNA-Gene-Network was built to underlying that the key miRNAs potentially regulate the expression of genes related with the effects of IH in adipocytes. Two miRNAs (miR-182-5p, miR-30c-2-3p) were found to have more conspicuous effects and might have main regulatory effects in the network since their degrees were more than 10 (Figure 3C). The significant pathways were determined in consistent with the functions and linking of DE genes based on KEGG database. The miRNA-pathway-network was constructed on the basis of interaction between vital pathways and genes and the interaction between miRNA expressions and gene expressions (Figure 3D).

Key differentially expressed miRNA (miR-182-5p and miR-30c-2-3p) were selected for qRT-PCR validation. As shown in Figure 6, the expression levels of miR-182-5p and miR-30c-2-3p were decreased in the IH-treated group compared to the controls (Figures 3E,F). The tendencies exhibited were approximately accordance with that in miRNA sequence in the results of qRT-PCR.

### Bioinformatics analysis of verified miRNAs and the effect from intermittent hypoxia-exposed fat cells on pathway

**FIGURE 4:**
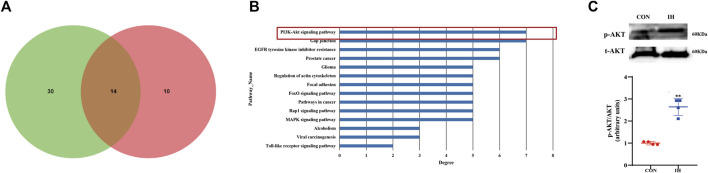
Bioinformatics analysis of verified miRNAs and the effect from IH-exposed fat cells on pathway. **(A)** The overlapped predicted pathways of mir-182-5p and mir-30c-2-3p is showed by venn diagram. **(B)** KEGG Pathway analysis of 2 differentially expressed miRNAs. **(C)** The protein expression of p-Akt in the effect of IH (**p value < 0.05).

Venn map showed that miR-182-5p and miR-30c-2-3p had over 14 target pathways predicted by pathway analysis (Figure 4A). PI3K/Akt signal pathway was the top pathways in which downregulated miR-182-5p and miR-30c-2-3p (Figure 4B). Considering the enriched function of differentially expressed miR-182-5p and miR-30c-2-3p was mainly concentrated in PI3K/Akt pathway, we explored the effect of IH-treated fat cells on PI3K/Akt pathway. Western blot showed that phosphorylated Akt were significantly upregulated in IH-treated group compared to the control groups (Figure 4C). The results showed PI3K/AKT pathway is a downstream target pathway of miR-182-5p and miR-30c-2-3p.

### Both miR-182-5p and miR-30c-2-3p are involved in proliferation and metabolism of adipocyte *via* PI3K-AKT pathway

**FIGURE 5:**
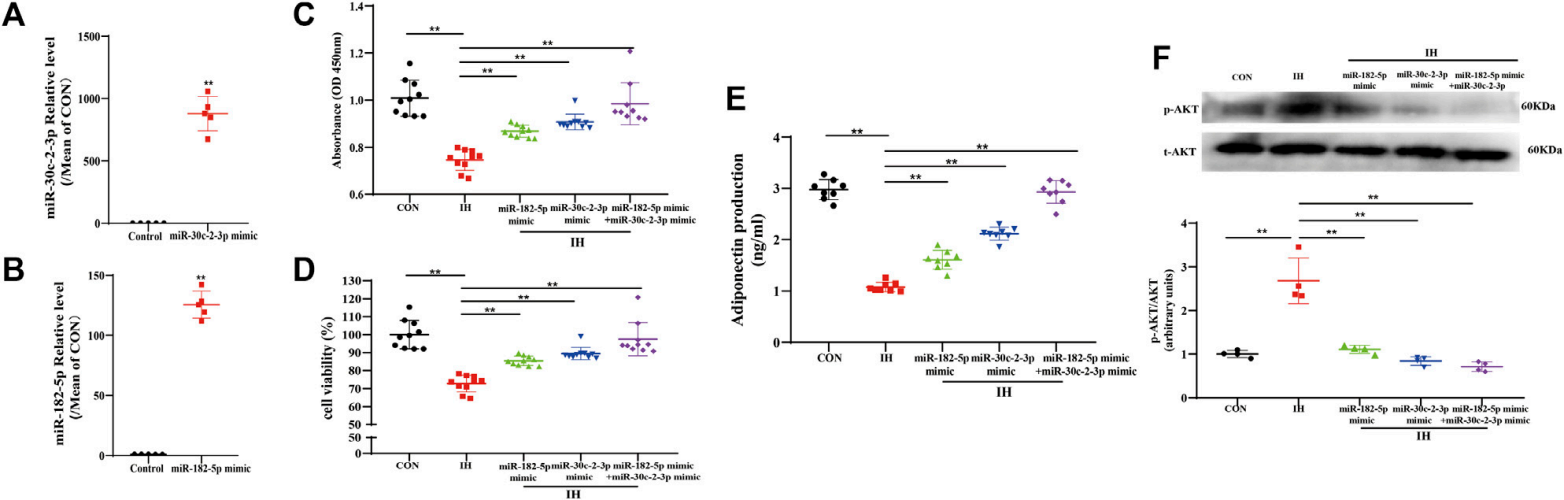
Both miR-182-5p and miR-30c-2-3p are involved in proliferation and metabolism of adipocyte via PI3K-AKT pathway. The levels of miR-30c-2-3p **(A)** and miR-182-5p **(B)** in adipocytes. **(C,D)** CCK8 assay demonstrated that miR-182-5p and miR-30c-2-3p overexpression promoted adipocytes growth and viability. **(E)** Concentrations of adiponectin from the culture supernatant in adipocytes were determined using ELISA kits. **(F)** The protein expression of p-Akt in the IH-induced adipocytes of transfecting miR-182-5p and miR-30c-2-3p mimic. *n* = 4–10/group, **p < 0.01 vs. IH group.

To clarify the functional role of miR-182-5p and/or miR-30c-2-3p in adipocytes metabolism regulated by IH, we succeeded in establishing a stable overexpression system of miR-182-5p or miR-30c-2-3p through the miRNA mimics transfection of 3T3L-1 cells (Figures 5A,B). Then we detected the proliferation and viability of adipocytes *via* CCK8 assay and the secretion of APN in adipocytes supernatant by ELISA. As shown in Figures 5C,D, compared with the control group, IH inhibited cell proliferation, resulting in decreased cell viability. However, stable transfections of mimic miR-182-5p or/and miR-30c-2-3p promoted proliferation and reversed the decrease in cell viability caused by IH. In addition, we demonstrated that the inhibition of adiponectin secretion in adipocytes induced by IH could be rescued by miR-182-5p or miR-30c-2-3p. Besides, the combination of the two miRNAs exhibits a stronger promotion on adiponectin secretion of adipocytes than single transfection (Figure 5E). To further explore the relationship between miR-182-5p/miR-30c-2-3p and PI3K-AKT pathway, PI3K/AKT pathway-related proteins expressions were detected by westernblot. Compared with the NC group, p-AKT expression was decreased in miR-182-5p and/or miR-30c-2-3p group in adipocytes treated by IH (Figure 5F). Overall, upregulation of miR-182-5p and miR-30c-2-3p mediated adipocytes metabolism *via* PI3K/AKT pathway.

## Discussion

The prevalence of obstructive sleep apnea (OSA) is increasing. OSA is a metabolic disease, and clinical studies have found that OSA with IH as the main clinical feature is closely related to a number of metabolic diseases such as obesity, type 2 diabetes (T2D) obesity and so on (Loffler et al., 2020). At the same time, some basic research reports show that OSA is related to metabolic dysfunction. IH exposures mimicking OSA induce changes in gut microbiota, increase gut permeability, and alter plasma exosome cargo, the latter inducing adipocyte dysfunction (increased insulin resistance) (Khalyfa et al., 2021). It has been reported that upregulation of proadipogenic markers and downregulation of antiadipogenic markers in line with smaller size of adipocytes were found in IH-exposed subcutaneous adipose tissue of Sprague-Dawley rats (Wang et al., 2018). In addition, exosomes from fasting morning plasma samples from obese adults with OSA induced significant increases in adipocyte lipolysis that were attenuated after CPAP (Khalyfa et al., 2018). All the above reports confirmed OSA/IH induces the disorder of adipocytes (the body’s metabolic regulatory organ). Several studies have focused on the effects of OSA on dyslipidemia and lipid metabolism (Barros and García-Río,2019; Peng et al., 2021). These data suggest that IH induces hyperlipidemia, and results in the up-regulation of genes involved in lipid biosynthesis in the liver and adipose tissue, and increased lipoprotein secretion. Research into OSA-related miRNA and mRNA changes has focused on OSA with complications (Du et al., 2020). Together these studies demonstrated that IH facilitate adipocyte metabolism dysfunction. However, the key mRNAs and miRNAs leading to this pathological change are not clear. Therefore, in the current study, we illustrate for the first time IH-exposed molecular changes to adipocytes using high-throughput sequencing technology and demonstrate miR-182-5p and miR-30c-2-3p may be the key molecules in adipocyte disorder induced by IH.

Obstructive sleep apnea is a common clinical condition in which the throat narrows or collapses repeatedly during sleep, the direct consequences of the collapse are intermittent hypoxia and hypercapnia, and recurrent arousals, leading to secondary sympathetic activation, oxidative stress, and metabolic alterations (Patel, 2019). A large clinical data shows that metabolic alterations are independent risk factors in the cardiovascular consequences associated with OSA (Garbarino et al., 2018). It has reported that OSA severity correlated with the amount of visceral fat than to body mass index (BMI) (Giampá et al., 2022; Neeland et al., 2020). Many studies show that OSA affects the release of adipokines in the adipocyte tissues (Li et al., 2010; Gami et al., 2003). In our study, we analyzed the clinical baseline in 20 OSA patients and 20 healthy, the data shows that OSA patients had a higher BMI compared to healthy group. The result suggests that OSA may induce adipocyte metabolism.

Marked advances in high-throughput sequencing technology allow us to identify the expression profiles of mRNA in tissues or organisms. We have demonstrated that OSA is related to remarkable changes in plasma mRNA expression profiles of OSA patients. OSA was related to changes in the expression of 343 genes. The differential genes were 3035 in IH-induced adipocytes. By employing GO analysis, we found that lipid metabolic processes formed the most affected gene functional category in the IH-induced adipocyte model. This is consistent with finding of GO analysis in OSA patient plasma. Our study supports that OSA is related to adipocyte metabolism.

Based on our KEGG database, the enrichment pathway concentrated on oxidative phosphorylation, citrate cycle and pyruvate metabolism in differential genes from OSA patient plasma. However, the most critical pathway involved in the response to IH-induced adipocyte was the PPAR signaling pathway. The peroxisome proliferator-activated receptors are members of the nuclear receptor super-family. They act as transcription factors ligand-inducible and play important roles in lipid metabolism and glucose (Wang et al., 2016). Hypoxic treatment was previously shown to aggravate endothelial damage by down-regulating the PPARγ pathway in the leptin receptor deficient db/db mice, which are used to model obesity and insulin resistance (Legchenko et al., 2018). Lung macrophage cells from obese patients with OSA have been shown to express a 2-fold reduction in PPARγ mRNA (Sharma et al., 2012). Our results indicated that IH resulted in disordered adipocyte PPAR signaling.

Many discoveries are critical as miRNA-based therapies hold great promise for the treatment of metabolic disorders (Huang, 2018). Previous studies have suggested that miRNA-21 attenuates post-myocardial-infarct remodeling and heart failure in mice (Bejerano et al., 2018). Meanwhile, serum levels of miR-221, miR-28-3p (Guay et al., 2019), miR-142-3p, miR-486-3p, and miR-486-5p (Meerson et al., 2019) can be used to assess risk for type-2 diabetes. We have demonstrated that IH is related to remarkable changes in adipocyte miRNA expression profiles. IH was related to changes in the expression of 59 miRNAs. 68 differentially expressed mRNAs were obtained in plasma from OSA patient and IH-induced adipocyte model. At the same time, we observe that 68 differential genes could be connected to 49 reciprocally expressed miRNAs. Given these findings, IH induce the alteration of miRNAs expression profile in adipocyte; further verification of the relationship between these miRNAs and IH is therefore warranted.

Our results also showed that mir-182-5p and mir-30c-2-3p were downregulated by PCR in the IH group. MicroRNAs (miRNAs) are small noncoding RNAs that are participated in post transcriptional vanishing of target genes *via* either miRNA degradation or translational inhibition or both. Dysregulation of known miRNAs has been related to metabolic diseases (Ji and Guo, 2019). It was previously reported that whole blood levels of mir-182-5p can predict diabetes (Weale et al., 2020). miR-182-5p has also been reported to play an important role in atherosclerosis by inhibiting oxidative stress and apoptosis through the oxidation of low-density lipoprotein (Zhao et al., 2020). miR-182-5p from liver tissues has been shown to improve glucose levels in mice and has been linked to the pancreatic (Weale, et al., 2020), liver (Cao et al., 2018), and breast cancers (Sang et al., 2019). Until now, miR-30c-2-3p has not been linked to adipocyte metabolism. Reports demonstrated that upregulation of miR-30c-2-3p indicated a poor survival in gastric cancer (Fan et al., 2022). miR-30c-2-3p promotes the development of lung adenocarcinoma by targeting MYBL2 and regulating CELL CYCLE Signaling Pathway (Xu et al., 2021). Since there are generally few studies on miR-30c-2-3p, more research needs to be warranted. Given these findings, miR-182-5p may prove to be one of the vital key and regulatory microRNAs related to the fat cells metabolism induced by the effects of IH; further verification of the relationship between these miRNAs, IH, and adipocyte metabolism is therefore warranted.

In this study, there are several limitations. Firstly, although we used transcriptomic sequencing combined with bioinformatics technology to screen for differentially expressed mRNAs and miRNAs in adipocytes under OSA with IH as the main pathological feature, we did not explore too many mechanisms. Therefore, in the further study, we will identify the target genes of miR-182-5p and miR-30c-2-3p, and analyze the relationship between target genes and PI3K/AKT pathway or IH/OSA. Secondly, we are sorry that human samples were not used for miRNA sequencing in this study. According to the requirements of RNA sequencing, only samples meeting the conditions of RNA Integrity Number (RIN) ≥ 7.5 and total RNA ≥ 2 μg can be used to the subsequent analysis, including mRNA/miRNA purification, cDNA library construction and transcriptome/small RNA sequencing. However, due to ethical issues, the amount of blood collected from patients and healthy controls was limited (up to 2 ml). The amount of RNA extracted from human plasma was also too small to meet the requirements of both mRNA and miRNA sequencing. Therefore, we only performed the mRNA sequencing in human samples.

In conclusion, we got several innovative discoveries. First, our study determined that an mRNA expression profile is related to remarkable changes in plasma from OSA patients. IH may cause important changes in mRNA and miRNA expression profiles in adipocytes. Second, our data supports that miR-182-5p and miR-30c-2-3p may play a crucial role in IH-induced adipocyte metabolism. Further research is needed to verify the target genes of miR-182-5p and miR-30c-2-3p and the underlying responses of adipocytes to IH.

## Data Availability

The datasets presented in this study can be found in online repositories. The names of the repository/repositories and accession number(s) can be found below: NCBI (accession number GSE210021, PRJNA863924 and PRJNA863063).
