# Supplementary material for: Potential regulatory role of miRNA and mRNA link to metabolism affected by chronic intermittent hypoxia
Source: Front Genet. 2022 Sep 6;13:963184. doi: 10.3389/fgene.2022.963184 (PMC9485438; doi:10.3389/fgene.2022.963184)
Supplement: Supplementary file 1 [file Table1.DOCX]

SUPPLEMENTAL MATERIAL

**Supplemental Table 1. The baseline characteristics of the patients and controls**

|  | **Control group(n=3)** | **OSA group(n=3)** | ***p*** | |
| --- | --- | --- | --- | --- |
| **Age(Year)** | 54±8 | 48±15 | | 0.557 |
| **Gender(Male/Female)** | 3/0 | 3/0 | |  |
| **BMI** | 21.1±1.6 | 26.53±1.33 | | 0.0343 |
| **AHI** | 3.23±0.75 | 50.03±1.89 | | <0.001 |
| **SpO_2_** | 89.25±1.56 | 68.33±7.51 | | 0.009 |
| **TC(mmol/L)** | 4.54±0.71 | 4.89±1.24 | | 0.686 |
| **TG(mmol/L)** | 0.77±0.24 | 2.69±0.95 | | 0.027 |
| **LDL(mmol/L)** | 2.48±0.39 | 4.08±1.05 | | 0.068 |
| **GLU(mmol/L)** | 4.94±0.65 | 6.62±0.95 | | 0.065 |

**OSA: obstructive sleep apnea. Values are expressed as mean±SD.**
